# An Overview of Drug Resistance in Protozoal Diseases

**DOI:** 10.3390/ijms20225748

**Published:** 2019-11-15

**Authors:** Rita Capela, Rui Moreira, Francisca Lopes

**Affiliations:** Instituto de Investigação do Medicamento (iMed.ULisboa), Faculdade de Farmácia, Universidade de Lisboa, Av. Prof. Gama Pinto, 1649-003 Lisboa, Portugal; rmoreira@ff.ulisboa.pt (R.M.); fclopes@ff.ul.pt (F.L.)

**Keywords:** protozoan, drug resistance, malaria, leishmaniasis, trypanosomiasis

## Abstract

Protozoan diseases continue to be a worldwide social and economic health problem. Increased drug resistance, emerging cross resistance, and lack of new drugs with novel mechanisms of action significantly reduce the effectiveness of current antiprotozoal therapies. While drug resistance associated to anti-infective agents is a reality, society seems to remain unaware of its proportions and consequences. Parasites usually develops ingenious and innovative mechanisms to achieve drug resistance, which requires more research and investment to fight it. In this review, drug resistance developed by protozoan parasites *Plasmodium*, *Leishmania*, and *Trypanosoma* will be discussed.

## 1. Introduction

Protozoal diseases have an enormous health, social, and economic impact and contribute significantly to the burden of infectious diseases worldwide. Malaria (*Plasmodium* spp.), the several forms of cutaneous and visceral leishmaniasis (*Leishmania* spp.), African sleeping sickness (*Trypanosoma brucei*), Chagas’ disease (*Trypanosoma cruzi*), amoebic dysentery (*Entamoeba* spp.), and toxoplasmosis (*Toxoplasma* spp.) are severe diseases that threaten human lives of nearly one- sixth of the world population [1]. 

Protozoal disease burden affects mainly tropical and subtropical regions, but environmental changes and ecological modifications, due to both natural phenomena and human intervention, have displayed and can be expected to continue to display a significant influence on the emergence and proliferation of these infections in high-income countries. 

The noteworthy impact of human protozoan infections has been augmented by the lack of effective vaccines and safe and affordable drugs, for prevention and treatment of these diseases. Unfortunately, the usefulness of available drugs is being increasingly threatened by the development of parasite drug resistance. The need for new antiprotozoal drugs drives research across the world and requires innovative strategies to ensure a sustainable discovery of lead compounds.

In this review we will focus on drug resistance in protozoa, mainly in malaria and diseases caused by *Leishmania* and *Trypanosoma* spp. 

## 2. The Triangle Relationship: Parasitic Protozoa, Host, and Drug Resistance

Protozoa are microscopic unicellular eukaryotic organisms found worldwide. More than 65,000 species of protozoa have been described, most of which are free-living organisms. These species have a relatively complex internal structure and carry out complex metabolic activities. [2]. The developmental stages of the parasites generally consist of feeding trophozoites, either intracellularly (within host cells) or extracellularly (in hollow organs, body fluids, or interstitial spaces between cells). The transmission between hosts, can be direct, fecal-oral, vector-borne, and predator-prey transmission [3,4]. The life cycle of protozoa also have dormant cysts and in this form, the protozoa can survive in extreme conditions, without oxygen, water, or nutrients for a long period of time.

The armamentarium of antiprotozoal drugs is limited, and the effectiveness of these drugs is being diminished by resistance development, as in the case of widespread resistance to some of the most effective drugs ever developed as: Chloroquine in malaria, metronidazole in anaerobic parasites, sulfonamide in *Toxoplasma gondii*, and diloxanide for intestinal protozoan [5,6,7]. The emergence and spread of drug resistance, combined with a lack of effective vaccines, is a major challenge to control protozoan infections. Another important feature is the fact that most of the studies on drug resistance in protozoa have been done with laboratory strains under conditions that do not mimic the normal parasite-host relation.

In addition, host health influences the spread of infection. Individuals whose defense systems are able to control, but not to eliminate, a parasitic infection, become carriers and constitute a source of infection for others. In geographic areas of high incidence, well-tolerated infections are often not treated in a way to eradicate the parasite because eradication would lower the individual’s immunity to the parasite and result in a high probability of reinfection.

## 3. Antimalarial Drug Resistance

Malaria remains a major public health problem in most of the tropical world. More than 200 million cases and 435,000 deaths were estimated worldwide in 2017. Importantly, 11 countries contribute for approximately 70% of estimated malaria cases and deaths globally. From these, 10 belong to sub-Saharan Africa and India [8]. Among these countries, only India reported progress in reducing its malaria cases in 2017 compared to 2016 [8]. 

**Figure 1:**
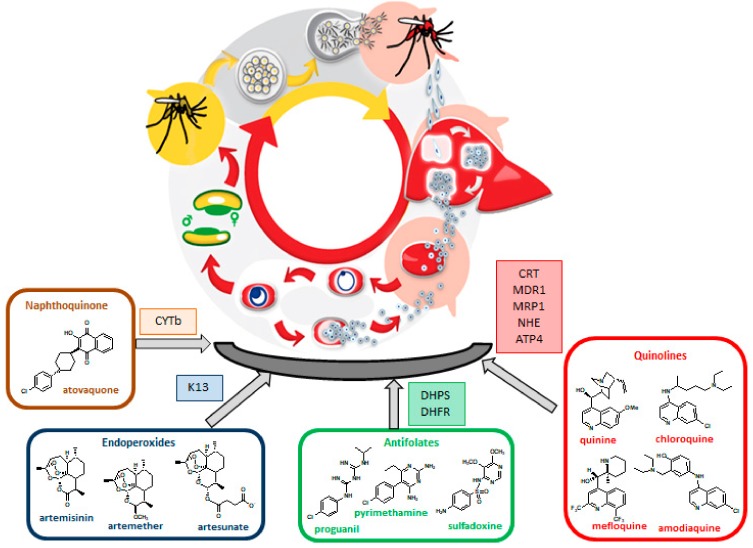
The major classes of antimalarials and the corresponding target mutations responsible for resistance. (CYTb—Cytochrome b; K13—kelch 13 protein; DHPS—dihydropteroate synthetase; DHFR—dihydrofolate reductase; CRT—chloroquine resistance transporter; MDR1—multidrug resistance protein 1; MRP1—multidrug resistance-associated protein 1; NHE—Na^+^/H^+^ exchanger protein; ATP4—ATPase sodium efflux pump).

*Plasmodium falciparum* and *Plasmodium vivax* are responsible for the largest number and for the most severe cases of the disease and also for the most drug-resistant infections [9]. The malaria parasite exhibits a complex life cycle involving an *Anopheles* mosquito and a vertebrate host. When an infected female mosquito bites a human, *Plasmodium* sporozoites are injected in the bloodstream and travel to the liver, invading hepatocytes. Here, parasites evolve to hepatic schizonts producing several thousands of merozoites that will be released in the bloodstream. Upon erythrocyte invasion, *Plasmodium* parasites undergo asexual replication forming mature schizonts whose rupture releases new merozoites which invade new erythrocytes. Clinical symptoms appear during this stage. Some parasites differentiate into gametocytes that, when ingested by the mosquito in a new blood meal, evolve to gametes. Gamete fusion in the insect midgut produces a zygote, which develops to a motile ookinete, traversing the gut wall, producing sporozoites that will be injected in a new human host by the insect bite completing the life cycle (Figure 1). *P. vivax* and *Plasmodium ovale* can develop dormant forms in the liver stage responsible for relapses of the disease. In *P. falciparum* infection, the ability of parasites to sequester in the microvasculature of several organs, including the brain, is a major cause of disease severity, and of a fatal outcome [10,11].

Available antimalarial drugs can be divided into multiple classes (Table 1). 

Table 1 resumes the antimalarials currently in use, their mode of action and the mechanism of resistance. Almost all drugs in clinical use act primarily against the intraerythrocytic development of *Plasmodium* parasites. For the treatment of *P. falciparum* malaria, the most important drugs are developed to target either the food vacuole of ring-stage and trophozoites of blood-stage malaria or the enzymes involved in the trophozoite folic acid biosynthesis pathway [14]. However, to meet the goal of malaria eradication, drugs that prevent parasite transmission and eliminate the asymptomatic and hepatic forms need to be developed [15,16,17]. The global strategy for malaria comprises three major pillars, which are: (a) Ensure universal access to malaria prevention, diagnosis and treatment; (b) accelerate efforts towards elimination and attainment of malaria-free status; (c) transform malaria surveillance into a core intervention. These three pillars are supported with two important elements: Innovation and research and a strong enabling environment [18]. 

Malaria control and elimination are threatened by the development and spread of resistance to drugs including artemisinins and partners on ACTs (Artemisinin Combination Therapy), the first line treatment recommended by WHO. Currently, there is documented resistance to antimalarial drugs in three of the five malaria species that affect humans: *P. falciparum*, *P. vivax,* and *Plasmodium malariae*. In addition, intensification of antimalarial drug resistance can also be ascribed to cross resistance between drugs of the same chemical family or sharing similar modes of action [19]. Assessment of drug-resistance biomarkers and therapeutic efficacy studies in malaria endemic regions will help to detect the resistant parasite, and also to understand the degree and extent of resistance associated with a particular population [20]. The most representative classes of antimalarials and the corresponding targets mutations responsible for resistance are presented in Figure 1.

### 3.1. Resistance to Quinolines

Chloroquine (CQ), a 4-aminoquinoline, was the gold standard for the treatment of malaria during the 1960s and 1970s. The emergence of *Plasmodium falciparum* resistant to CQ in Southern Asia was a major stumbling block in the global control of malaria [21]. Although CQ continues to be a first-line treatment for *P. falciparum* malaria in Central America, the rise of CQ resistance contributed to a worldwide increase in malaria-related mortality [8]. 

CQ and related 4-aminoquinolines act through the inhibition of hemozoin formation in the digestive vacuole, during ring and trophozoite stages [22]. Dicationic CQ interferes with the parasite detoxification process by inhibiting the transformation of heme (toxic to parasite) into hemozoin (non-toxic to parasite) [23,24]. 

#### Resistance-Associated Mutations

There is strong evidence that resistance to antimalarial drugs is associated to parasite genetic factors. Simple, double, or quadruple mutations in different genes enable the parasite to resist to the antimalarial drugs. Mutations in *Pfmdr1*, *Pfcrt*, *Pfmrp,* and *Pfnhe1* genes have been fixed in several parasite populations and, since they confer drug resistance, this facilitates their dispersion [25]. 

*Pf*crt and *Pfmdr1*—The *P. falciparum* genome encodes multiple predicted transporters. In the case of malaria, polymorphism in plasmodial proteins transport impacts drug sensitivity [26]. CQ resistance results from multiple mutations in *Pf*CRT (*P. falciparum* chloroquine resistance transporter). These mutations enable *Pf*CRT to efflux CQ out of the parasite digestive vacuole, thereby preventing CQ from binding to heme and inhibiting its detoxification [24]. *Pf*crt gene has 13 exons, localized on chromosome 7, encoding for a 424 amino acid transmembrane protein. *Pf*CRT protein belongs to the drug/metabolite transporter superfamily with 10 putative transmembrane domains spanning the digestive vacuole membrane of the parasite. Mutation in *Pf*crt gene plays a significant role in determining the CQ resistance and its phenotype [27]. The K76T mutation is the primary determinant of CQ resistance and susceptibility [28]. The positively charged lysine residue is replaced by neutrally charged threonine residue at 76^th^ position, which could allow the efflux of diprotonated CQ out of the digestive vacuole by active transport. Common mutations in other regions (C72S, M74I, N75E, A220S, Q271E, N326S, I356T, and R371I) also confer resistance, but only in association with the K76T mutation [20]. Variation in the *Pf*CRT protein also influences antimalarial drug susceptibility and resistance to quinine, amodiaquine, and lumefantrine. CQ shows cross-resistance with amodiaquine and quinine that is mainly mediated by 76T, whereas lumefantrine exhibits an inverse cross-resistance having reduced susceptibility in association with wild-type K76. The *Pf*CRT mutations at codons 72 to 76 confer higher resistance to CQ and medium level of AQ resistance in Southeast Asia and Africa, whereas linked with greater AQ resistance in South America. Thus, K76T mutation in *Pf*CRT protein is a potent molecular marker for antimalarial drug resistance, depending on their previous use in the region [20] 

In addition to *Pf*CRT, there is also the important *P. falciparum* multidrug resistance protein 1 (*Pf*MDR1) [24]. *P. falciparum* multidrug resistance-1 (*Pf*mdr1) gene encodes the P-glycoprotein homolog and impacts on sensitivity to multiple antimalarial drugs. In humans, P-glycoprotein polymorphisms are associated with resistance to cancer drugs. In *P. falciparum*, the function of the *Pf*mdr1 product is unknown, but the protein localizes on the membrane of the food vacuole, the site of action of a number of drugs, suggesting that it is a drug transporter [26]. *Pf*mdr1 gene is located on chromosome 5, has one exon, and encodes for the *Pf*MDR1 protein, with 1419 amino acids, which is a transmembrane protein located in the digestive vacuole. *Pf*MDR1 contains two domains, each one consisting of six helical transmembrane domains and a nucleotide binding fold region that act as a site for ATP binding, and belongs to the ATP-binding cassette (ABC) superfamily. Polymorphism, amplification, and variation in mRNA expression level of the *Pf*mdr1 gene have been involved in resistance to various antimalarials and emergence of multidrug resistance parasites [29]. Mutations in *Pf*mdr1 gene at N86Y, Y184F, S1034C, N1042D, and 1246Y, have been reported to affect the drug susceptibility to CQ, quinine, mefloquine, lumefantrine, and artemisinin. *Pf*MDR1 mutations at N86Y and N1042D position have been associated with amodiaquine resistance [20,30]. Data suggest that changes in *Pf*mdr1 sequence or copy number, alter transport of multiple drugs in or out of the parasite food vacuole, with individual polymorphisms leading to opposite effects on different drugs [19]. 

*Pf*mrp1—*P. falciparum* multidrug resistance-associated protein (*Pf*MRP) is a member of the ABC transporter superfamily, localized principally in the parasite plasma membrane [31]. *Pf*mrp gene has one exon, is located on chromosome 1, and encodes an 1822 amino acids protein. It is predicted to have two nucleotide binding domains and two membrane-spanning domains, each one consisting of six helical transmembrane domains. *Pf*MRP helps in the transport of organic anionic substrates such as oxidized glutathione, glucuronate, sulfate conjugates, and also in drug transport. Two mutations at positions Y191H and A437S in *Pf*MRP were associated with CQ and quinine resistance [26]. Genetic knockout of *Pf*mrp gene in the resistant parasite, showed high sensitivity to various antimalarial drugs such as CQ, quinine, primaquine, and artemisinin. It has also been hypothesized that *Pf*MRP protein effluxes various metabolites and drugs out of the parasite in association with other transporters [20].

In addition to *Pf*MRP1, *Pf*MRP2, a full transporter that belongs to the ABC C family, and *Pf*MDR5, a half transporter, that belongs to the ABC B family, were also described. All three ABC transporters proteins are located on the plasma membrane in all asexual erythrocytic stages of *P. falciparum*. This localization emphasizes the putative role of drug exporters of these ABC family members. As *Pf*MRP1, *Pf*MRP2, and *Pf*MDR5 might have a similar role in the efflux of glutathione, chloroquine, and quinine and thereby broadening the capacity of the parasite to extrude toxic compounds [31,32,33]. 

*Pf*nhe and *Pf*ATP4—The *P. falciparum* Na^+^/H^+^ exchanger (*Pf*NHE) is a transmembrane protein located in the plasma membrane of the parasite with 1920 amino acids and predicted to have 12 transmembrane domains. The role of *Pf*NHE was not fully understood, but it was hypothesized that it is involved in active efflux of protons to maintain pH 7.4 within the parasite, in response to acidification by anaerobic glycolysis, the parasite’s main source of energy [20].

*Pf*NHE contains three microsatellite regions and the increase of DNNND repeat number in microsatellite ms4670 has been associated with decreased quinine susceptibility in some studies. However, three mutations at 790, 894, and 950 codons and polymorphism in the microsatellite region (msR1 and ms3580) showed no association with quinine resistance. The varying results concerning the association of *Pf*NHE mutations with quinine resistance indicate either that another gene, in close physical proximity of *Pf*nhe, could be responsible for the reduction in quinine susceptibility or, that *Pf*NHE requires other additional genetic factors for mediating quinine resistance. Due to that, the DNNND repeat number can only be a valid marker for quinine resistance in some genetic backgrounds, but not for all [34,35]. Thus, repeat polymorphism in *Pf*nhe1 gene may be used as a valid genetic marker to determine the quinine resistance in some regions, and resistance to quinine can also be mediated by other genetic markers such as *Pf*crt, *Pf*mrd1, and *Pf*mrp [20].

Another ATPase sodium efflux pump is the *P. falciparum* plasma membrane protein *Pf*ATP4. The *Pf*ATP4 multidrug resistance mutation G223R was found in Africa by genetically analyzing 2640 *P. falciparum* blood stage isolates (the MalariaGen *Pf*3k resource). This mutation increases approximately eight-fold the *Pf*ATP4 IC_50_ of spiroindolones (KAE609 and KAE678) and aminopyrazoles (GNF-*Pf*4492). It is postulated that the G223R mutation may be a consequence of the drug resistant Southeast Asian Dd2 genotype becoming more dominant in Africa. The presence of this mutation has important policy implications for the eventual general deployment of the spiroindolone KAE609 (Cipargamin), which is currently undergoing stage 2 clinical trials and initiate parenteral administration in humans in 2019 [26,36].

### 3.2. Resistance to Antifolates

Proguanil was the first reported antimalarial antifolate agent and was discovered by Imperial Chemical Industries during the Second World War. After this discovery, studies demonstrated that, in fact, proguanil is a prodrug and metabolizes to its triazine form cycloguanil, an inhibitor of the parasite dihydrofolate reductase (DHFR). Similarly to proguanil, chlorproguanil is metabolized to chlorcycloguanil, the active metabolite. This antifolate was combined with dapsone for treatment of uncomplicated malaria. However, both of these drugs have been discontinued in 2008 following increasing evidence of toxicity of dapsone in the form of haemolysis in patients with G6PD deficiency [37]. 

Pyrimethamine is a 2,4-diaminopyrimidine synthesized and tested in the late 1940s as an analogue of folic acid for treatment of tumors. The structural similarity with proguanil, led to the hypothesis that these compounds could have antimalarial activity. All these antifolates have a higher affinity of binding with *P. falciparum* than human DHFR [37].

The discovery that sulfa drugs block the synthesis of folate, led to the use of this class of compounds as antimalarial agents since the parasites rely on de novo synthesis of folate. These sulfa drugs acts as dihydropteroate synthetase (DHPS) inhibitors and belong to two families: sulphonamide (sulfadoxine) and sulphone (dapsone). The interest in this class of antifolates was fostered when it was demonstrated that they synergized with anti-DHFR [37,38].

#### Resistance-Associated Mutations

*P. falciparum* and *P. vivax* rapidly developed resistance against antifolates associated to mutations in genes encoding for DHFR and DHPS. These mutations have been used as markers for tracking resistance to sulfadoxine-pyrimethamine (SP) [26,39].

*Pf*dhps—The *Pf*dhps gene is located on chromosome 8, contains three exons and encodes for *Pf*DHPS protein, which consists of 706 amino acids. *Pf*DHPS enzyme catalyzes the reaction to obtain the folate precursor that is essential for the synthesis of pyrimidine in the parasite. Five mutations in the *Pf*DHPS protein (S436A/F, A437G, K540E, A581G, and A613T/S) are known to be involved in sulfadoxine resistance in *P. falciparum*. Mutation at 436, 581, and 613 codons are associated with higher level of resistance, whereas mutation at 437 and 540 contribute to a low level of resistance with modulation effects in association with other mutation in *Pf*DHPS. Since the antimalarial drug resistance as monotherapy has emerged, sulfadoxine is always provided in combination with pyrimethamine, and resistance to SP have been associated with point mutation in both *Pf*dhfr and *Pf*dhps gene [20,28,40,41].

*Pf*dhfr-ts—The *Pf*dhfr-ts gene has one exon located on chromosome 4 encoding for *Pf*DHFR protein with 608 amino acids in length. It is a bifunctional enzyme involved in two main folate metabolic activities: The biosynthesis of dTMP by thymidylate synthase activity and the reduction of dihydrofolate into tetrahydrofolate by dihydrofolate reductase activity. The key S108N mutation in *Pf*DHFR, introduces a larger side-chain amino acid that sterically interferes with the binding of inhibitors with a rigid pCl-phenyl side chain such as pyrimethamine and cycloguanil [42]. Antifolate resistance is amplified when the S108N mutation is accompanied by additional mutations in the inhibitor binding region of *Pf*DHFR including A16V, N51I, C59R, and I164L. Double mutation A16V and S108T in *Pf*DHFR is linked with the resistance of *P. falciparum* to cycloguanil [20,28,40,41,42].

### 3.3. Resistance to Artemisinin 

Artemisinin (ART), a sesquiterpene lactone bearing a peroxide group was discovered in 1975. The peroxide group is an essential factor for antimalarial activity. However, the poor solubility of ART in water or oil and the high rate of parasite recrudescence led to the discovery of ART derivatives. Dihydroartemisinin (DHA), artemether, artesunate, and arteether were developed and showed better efficacy, tolerability, and oral bioavailability than artemisinin, as well as minimal adverse effects. Artemisinin and derivatives are short-acting antimalarial drugs which have been shown to produce rapid relief from clinical symptoms and rapid clearance of the parasite from the peripheral blood. Since 1980s, millions of malaria patients in the world (mainly in China, Southeast Asia, and Africa) were saved by administration of ART, ART derivatives, and their combinations [43,44,45].

The proposed mechanism of bioactivation of artemisinin derivatives involves the cleavage of the endoperoxide bridge by a source of Fe^2+^ or heme. This cleavage results in the formation of oxy-radicals that rearrange into primary or secondary carbon-centered radicals. These reactive intermediates are proposed to alkylate proteins and form adducts with essential parasite macromolecules that result in the rapid death of the parasite [44,46,47]. 

The increasing problem of resistance development in the treatment of malaria led to recommendation by WHO since 2012, to use Artemisinin-based Combination Therapies (ACTs), where artemisinin and derivatives (artesunate, artemether, or DHA) are combined with a longer-acting antimalarial that has a different mode of action. ACT is used for the treatment of malaria in areas where *P. falciparum* is the predominant infecting species [48]. WHO currently recommends five ACTs for use against *P. falciparum* malaria, which are: Artemether+lumefantrine; artesunate+amodiaquine; artesunate+mefloquine; artesunate+SP and dihydroartemisinin+piperaquine. The choice of ACT should be based on the results of therapeutic efficacy studies against local strains of *P. falciparum* malaria [24,49]. 

#### Resistance-Associated Mutations

Resistance to ACTs emerged in 2008 in parts of Southeast Asia and continues to spread. Currently artemisinin resistance is prevalent in parts of Cambodia, the Laos People’s Democratic Republic, Myanmar, Thailand, and Vietnam [12,50]. Clinical artemisinin resistance manifests as an increased gametocytaemia and a slowed parasite clearance, which means that the infection can be solved using ACTs but with a substantially increased time required for treatment. However, this delayed clearance could contribute to the rise of multidrug resistance, as parasites have gained both reduced artemisinin sensitivity and resistance against partner drugs, such as piperaquine [21,51]. 

Mutations in kelch13 gene are usually considered as a genetic marker for artemisinin resistance [20,30] but some other polymorphisms have also been described as responsible for increased parasite clearance time [52]. This is the case of autophagy-related gene 18 (atg18) that encodes for the protein *Pf*Atp18. Patients with a mutation in *Pf*Atp18 (T38I) and treated with ACTs showed an increase in time clearance of parasites, in the absence of kelch13 polymorphism. One possible explanation is that this mutation can provide additional resistance against ARTs promoting a more efficient acquisition of nutrients through an autophagy-like pathway [53].

A long-term in vitro study for selection of DHA resistant parasites with two isolates from West Africa, showed consistently a mutation on the gene *Pf*coronin. This gene encodes for actin-binding protein Coronin and when this mutation was introduced into the parenteral parasites a reduction in ART susceptibility was observed [54].

*Pf*K13—K13 protein contains 726 amino acids and has one exon located on the chromosome 13. The C-terminal region of K13 protein has six kelch motifs consisting of beta sheets that fold into a propeller domain. Mutation in this region is predicted to disrupt the domain scaffold and alter its function. The kelch family proteins have diverse cellular functions, such as organizing and interacting with other proteins [20].

Recently, the point mutation in the propeller region of K13 protein has been identified as a key determinant for artemisinin resistance in *P. falciparum*. Nonsynonymous polymorphism at Y493H, R539T, I543T, and C580Y position observed in the kelch repeat region of K13 propeller domain have been associated with higher resistance to artemisinin. It was verified that M476I and D56V mutations increased the artemisinin resistance in Tanzania. In cultured and field isolates, mutation at these codons F446I, Y493H, P574L, R539T, and C580Y have contributed to a higher degree of resistance to artemisinin, and the frequency of C580Y allele mutation is higher and takes longer time for parasite clearance when compared to variation in other sites. In addition to the validated ART-resistant mutations Y493H, R539T, I543T, and C580Y, several novel nonsynonymous and synonymous candidate resistance mutations have been discovered in different countries. All the mutations have been reported to be associated with the clinical ART resistance [28,30,55]. 

### 3.4. Resistance to Atovaquone

The development of atovaquone as an antimalarial drug began when the outbreak of World War II caused substantial shortages in the supply of quinine. Intense research efforts in the USA led to several structurally diverse compounds, as hydroxynaphthoquinones. Quinones were reinvestigated in the 1980s with the aim to design a compound with antimalarial activity against *P. falciparum* combined with good metabolic stability in humans. Atovaquone, a hydroxynaphthoquinone, was the only one with a potency of 1 nM towards *P. falciparum* in vitro and not metabolized by human liver microsomes. The *trans* isomer of atovaquone is substantially more potent than the corresponding *cis* isomer [56]. 

Currently, atovaquone in combination with proguanil (Malarone) is recommended for the treatment of children and adults with uncomplicated malaria in non-endemic countries or in combination with artesunate and primaquine in areas where treatment failures of ACTs are problematic [12,56]. Malarone is also used as chemoprophylactic agent for preventing malaria in travellers and particularly military personnel whose experience of adverse effects with mefloquine is becoming increasingly recognized [56,57]. Atovaquone is a structural analogue of ubiquinone that selectively binds to the cytochrome b of *P. falciparum* (*Pf*CYTb), inhibiting the mitochondrial electron transport chain at the cytochrome bc1 complex and leading to the collapse of mitochondrial membrane potential. This mechanism is supplemented by the individual actions of proguanil and its metabolite, cycloguanil [56,57,58].

#### Resistance-Associated Mutations

Resistance to atovaquone arose rapidly in falciparum malaria when used as a single agent. The underlying reason for this phenomenon may be partially explained by pharmacodynamic/pharmacokinetic properties of atovaquone, combined with the effect of an increased mutation rate of mitochondrially encoded genes such as cytochrome b compared with nuclear encoded genes [56,59]. 

*Pf*cytb—The cytb gene encodes for a subunit of cytochrome bc1 complex, a mitochondrial membrane protein that catalyses the transfer of electrons across the inner mitochondrial membrane to maintain the electrochemical potential of the membrane. Cytochrome bc1 is predicted to have 10 putative helical transmembrane domains spanning the mitochondrial inner membrane of the parasite [20,60]. Resistance to atovaquone develops rapidly and is mediated by a number of mutations in the *Pf*cytb. The ubiquinol binding site, where the atovaquone competitively binds, is a highly conserved region of the protein and mutation in this region confers atovaquone resistance. Single mutations in *Pf*cytb (in particular leading to Y268S/C/N) cause atovaquone resistance both in vitro and in vivo [20,56,57]. However, episodes of treatment failure have been reported in the absence of the mentioned mutations [26].

### 3.5. Global Surveillance on Malaria Resistance

Currently, surveillance of anti-malarial drug resistance is done by any of three approaches: (a) In vivo studies to assess the efficacy of drugs in patients; (b) in vitro studies to evaluate parasite susceptibility to the drugs; and/or (c) molecular assays to detect validated gene mutations and/or gene copy number changes that are associated with drug resistance. The three methods are complementary, since they evaluate different aspects of resistance. WHO and several national and regional entities established an antimalarial drug resistance protocol in order to improve and maintain the antimalarial resistance surveillance networks worldwide [49].

## 4. Antileishmanial Drug Resistance

Leishmaniasis is a complex tropical/sub-tropical disease caused by more than 50 species of protozoa parasites of the genus *Leishmania*, 20 of which being pathogenic for humans. This disease is endemic in at least 98 countries and approximately one million new cases and 26 to 65 thousand deaths occur annually [61,62]. The parasites are transmitted between mammalian hosts by more than 90 female phlebotomine sandfly species. 

There are several different forms of the disease. (1) Visceral leishmaniasis (VL), also known as kala-azar, is the most serious form of the disease and it is fatal if untreated. (2) Post-kala-azar dermal leishmaniasis (PKDL), which usually appears six months to one year after apparent cure of VL. (3) Cutaneous leishmaniasis (CL), the most common, causes skin lesions, mainly ulcers on exposed parts of the body, leaving lifelong scars. (4) Mucocutaneous leishmaniasis (MCL), which leads to partial or total destruction of mucous membranes of the nose, mouth, and throat [63].

The life cycle of the parasite exhibits two morphological forms: Promastigotes in the gut of the sand fly vectors and amastigotes in macrophages of the mammalian host. The human stage of the life cycle begins when a parasitized female sand fly injects metacyclic promastigotes into the human body. The promastigote form suffers initially phagocytosis by the host’s macrophages of the skin where the parasite transforms into an amastigote, a non-flagellated form. From there, parasites disseminate and invade additional macrophages of the reticulo-endothelial system, and finally infiltrate the bone marrow, liver, and spleen. The multiplication of the parasite occurs inside the macrophages by binary fission. The macrophage lyses and the multiplication cycle continues when other hosts’ phagocytes are infected [64].

VL should be regarded as a state of long-term parasitism, since the parasites are not completely eradicated but rather remain in skin macrophages for lifetime. In the skin, *Leishmania* acts as a reservoir for the potential relapse of symptomatic VL. Fulminant reactivation of the infection is possible when T-cells immune responses are compromised, for example, due to post-transplant immunosuppressive therapy, use of immunomodulators, advanced age, or in HIV-infected patients [63].

**Figure 2:**
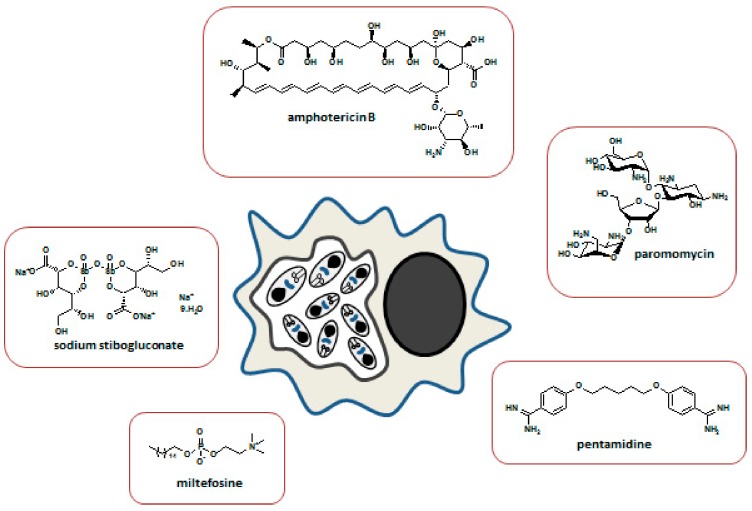
Intracellular amastigote form of leishmania parasite as the appropriate target for major antileishmanial drugs. Major drugs used in the treatment of leishmaniasis and their chemical structure.

Pentavalent antimonials (Sb(V)) were the standard treatment for leishmaniasis worldwide for almost 100 years. However, in the last 25 years their clinical action was compromised due to the widespread emergence of resistance, mainly in India, where failure rates of more than 60% have been observed in the treatment of VL caused by *L. donovani*. Second-line drugs, such as pentamidine (PMD) and amphotericin B showed emerging resistance and toxicity. New formulations of conventional drugs for leishmaniasis treatment, as well as innovative drugs, became available or are under investigation. The need for nontoxic and more effective drugs led to the development of liposomal amphotericin B in 1996, miltefosine in 2004 (considered as a third-line antileishmanial drug), and paromomycin in 2006, approved for the treatment of visceral leishmaniasis [65,66]. Drugs currently used for leishmaniasis treatment are presented in Table 2. Figure 2 depicts an intracellular amastigote form of *Leishmania* parasite as the appropriate target for major antileishmanial drugs.

Therapeutic solutions in low-income countries are limited mainly by high costs, but also due to toxicity issues and resistance emergence. Additionally, the control of the disease in these countries is further compromised by the emergence of HIV-VL co-infection [67]. 

The treatment of leishmaniasis fails in various countries mainly due to the emergence of resistance to pentavalent antimonials. Several mechanisms of antileishmanial resistance were identified in the last years. To overcome the phenomenon of resistance is fundamental to understand their molecular and biochemistry characteristics to achieve the design of novel drugs. 

Available antileishmanial drugs can be divided into multiple classes (Table 2). 

### 4.1. Resistance to Antimonials

The pentavalent antimonials sodium stibogluconate (SSG) and meglumine antimoniate (MA) are currently the only effective treatment with satisfactory clinical and microbiological results for all forms of leishmaniasis. These drugs showed severe side effects including pancreatitis, cardiac, and renal toxicity and can only be administered by injection, since there are no oral preparations accessible [68]. Both SSG and MA inhibit trypanothione reductase (TR), an enzyme considered crucial for parasite survival in the host. TR reduces trypanothione, used by the *Leishmania* tryparedoxin/tryparedoxin peroxidase system (TXN/TXNPx) to neutralize the reactive oxygen species produced by macrophages during the infection. In contrast to mammals, which use glutathione (GSH) as the fundamental key for redox defenses, trypanosomatid parasites rely on trypanothione as the main detoxifying system against oxidative damage [69]. 

It is generally accepted that pentavalent antimonials belong to the class of prodrugs, and that their conversion in vivo lead to the active/toxic trivalent antimonials Sb(III) responsible for *Leishmania* dead through apoptosis [70]. Acidic pH and slightly elevated temperature favor the reduction of Sb(V) to Sb(III). This reduction of antimonials can occur in macrophages as well as in the parasite. However, the ability of *Leishmania* to reduce Sb(V) to Sb(III) is stage-specific. Amastigotes can reduce Sb(V) to Sb(III), while promastigotes cannot, rendering amastigotes more susceptible to Sb(V) [71]. The incorrect use of antimonials by a majority of patients expose the parasites to drug pressure, leading to the development of tolerance, and eventually resistance, of the parasite to Sb(V) [72]. 

#### Resistance-Associated Mutations

Resistance to antimonials took a long time to emerge, suggesting that several mutations may be required to achieve a resistance phenotype. There are several in vitro mechanisms that can explain the observed antimonial resistance, but it should be noted that not always in vitro responsiveness necessarily translate to clinical resistance. The factors that can explain the emergence of resistance are: Reduction of drug concentration within the parasite, either by decreasing drug uptake or by increasing efflux of the drug; inhibition of drug activation; inactivation of active drug; and gene amplification [70].

Elevated intracellular thiol levels and overexpression of TXNPx are associated with high levels of Sb(III) resistance. In in vivo antimonial resistance, it was verified an inhibition of Sb(V) activation and a decreased uptake of the active form Sb(III) by amastigotes in the thiol metabolism. During this process there is a lower expression of the genes aquaglyceroporin 1, *γ*-glutamylcysteine synthetase, and ornithine decarboxylase, which are involved in the uptake of Sb(III) and metabolism of glutathione and trypathione, respectively. 

Another mechanism of antimonial resistance is the overexpression of the membrane-bound ATP-binding cassette (ABC) transporters on the surface of *Leishmania* parasites. This transport system modulates the efflux and intracellular accumulation of various drugs and thus, has a role in resistance development. The ABC transporters ABCI4 and ABCG2, can contribute to antimony resistance by the efflux of the drug as metal–thiol conjugates [73].

The multidrug resistance-associated protein 1 (MRP1) and the permeability glycoprotein (P-gp) in host cells are also upregulated by Sb-resistant *L. donovani* parasites, decreasing antimony influx, and consequently inhibiting intracellular drug accumulation [74].

Host membrane cholesterol is required for binding and internalization of *L. donovani* into macrophages. Cholesterol is an essential membrane lipid in higher eukaryotes and plays a vital role in the organization, dynamics, and function of membrane constituents. Statins, as lovastatin, are competitive inhibitors of HMG-CoA reductase, the rate-limiting enzyme in the cholesterol biosynthetic pathway. Lovastatin has been shown to inhibit the proteins MRP1 and P-glycoprotein in *L. donovani* allowing antimony accumulation and consequently reduces *Leishmania* cell growth and macrophage infection and leads to parasite death. In that way the class of statins reverse Sb resistance [74,75].

Flavonoids constitute a class of natural inhibitors of P-glycoprotein and related ABC transporters in *Leishmania*. Synthetic flavonoid dimmers have been used to reverse resistance to antimony drugs in *L. donovani* by increasing intracellular drug accumulation [74].

By functional cloning, to isolates drug resistance genes, the heat shock proteins (HSP70) was found to have a role in tolerance to antimony. An overexpression of HSP70 proteins was shown in cells in contact with antimony and Sb(III)-resistant mutants. *Leishmania* species transfected with HSP70 gene were more resistant to antimony, possibly due to increased tolerance of the cell to metals, allowing the cell to develop more specific and effective resistance mechanisms [76]. Recent studies also included HSP90 as a gene implicated in the emergence of resistance in *Leishmania* [77,78].

In summary, the overall phenomenon of antimonial resistance is multifactorial. Among clinical *Leishmania* isolates, several mechanisms of resistance to antimonials were detected [70,74,76].

### 4.2. Resistance to Pentamidine

The drug pentamidine, PMD, an aromatic diamidine, was used in 1937 for treatment of sleeping sickness. In 1949 was reported the first use in the treatment of antimony resistance cases of VL in India. PMD is administrated in cases of systemic CL caused by *L. guyanensis* and *L. panamensis* [66]. However, emerging resistance to PMD and additional side effects, as hypoglycemia, hypotension, fever, myocarditis, and renal toxicity were the main reason to discontinue this drug in India in the 1990s [79].

The precise mechanism of action of PMD in *Leishmania* is not well established, but some reports suggest that this drug disrupts the parasite mitochondrial inner membrane potential. The mitochondrial accumulation of pentamidine could also induce apoptotic death of the bloodstream form of *L. donovani* by inhibiting respiratory chain complexes I, II, and III, ROS generation and increase of cytosolic Ca^2+^, thus increasing cytotoxicity of the drug [80,81,82,83]. In addition, PMD can also target DNA topoisomerases (TOPs), which are essential in modulating DNA topology during replication, transcription, recombination, and repair. TOPI and TOPII from *Leishmania* parasites exhibit significant structural and biochemical variations from the corresponding human enzymes and perform critical functions in organizing the kinetoplast DNA network unique to these parasites. Studies have shown that PMD is selective for *Leishmania* TOPII [84].

PMD was also found to be a competitive inhibitor of arginine transport in *L. donovani* and a noncompetitive inhibitor of putrescine and spermidine transport in *L. infantum*, *L. donovani,* and *L. mexicana*. The drug enters both promastigote and amastigote forms of *L. mexicana* via a carrier-mediated process that recognizes the drug. However, the maximum velocity of uptake is substantially higher in amastigotes than in promastigotes [85,86].

#### Resistance-Associated Mutations

Pentamidine resistance in *Leishmania* parasites has been ascribed to mutations in several transporters. ABC transporters have been identified from different species of *Leishmania* and some members of this class have been well characterized and implicated in drug resistance [87]. The pentamidine resistance protein 1 (PRP1) is a member of the ABC transporters superfamily (ABCC7) which includes the P-glycoprotein (PGP) [73,88]. Aquaglyceroporin 2 (AQP2), a member of a family of surface channel proteins involved in the passive transport of water and small non-charged solutes across cell membranes, is the transporter responsible for resistance to high concentrations of pentamidine and melarsoprol in trypanosomes [89]. Studies need to be done, but there is the possibility that AQP2 mutation can also be responsible for pentamidine resistance in *Leishmania* parasites [74,90,91].

Not surprisingly, it was reported that the Ca^2+^ channel blocker and P-glycoprotein inhibitor verapamil is capable of inhibiting PMD efflux, leading to accumulation of PMD in resistant parasites [86]. To avoid the resistance to pentamidine in *Leishmania* parasites, flavonoid dimers were synthesized and exhibited a significantly higher reversing activity in pentamidine resistance in *L. enriettii*, due to an increased accumulation of the drug into the mitochondria. These synthetic flavonoids are reversal agents for overcoming PMD resistance in parasite *Leishmania*. The same dimers showed synergistic effect with quinacrine on reversing PMD resistance of *Leishmania* parasites [92,93].

### 4.3. Resistance to Amphotericin B

Amphotericin B (AMB), a polyene antibiotic originally extracted from the filamentous bacterium *Streptomyces nodosus* [76], is a second-line drug for the treatment of VL [70,94]. It is the best treatment against pentavalent antimonial refractive leishmaniasis in highly endemic regions such as Bihar state in India, and is also currently recommended by the National Program of Nepal for kala-azar treatment [94,95]. Despite being a highly effective antileishmanial drug, AMB displays significant secondary effects such as acute nephrotoxicity, implying hospitalization, and close monitoring of the patient during the four weeks course of treatment [65,68,79]. Another important additional disadvantage is the high cost of AMB. To avoid these drawbacks, a lipid-associated formulation, liposomal AMB (LAMB), have been developed with reduced toxicity and an extended plasma half-life, allowing the administration of a single infusion [68]. Oral and safer formulations of AMB for the treatment of leishmaniasis are being developed [94].

The mechanism of action of AMB may involve interaction with the membrane sterols, resulting in disorganization of the membrane and an increase in the permeability for protons and monovalent cations [94,96]. AMB could also affect the cells by its auto-oxidation and subsequent formation of free radicals. Cell damage generated by AMB might be associated with ion movement, oxidative effects, and the generation of reactive oxygen free radicals [97].

Amphotericin B is an increasingly important therapy for leishmaniasis, mainly due to the development of drug resistance to other treatments. Since there are few other drugs available, the potential for emergent resistance to AMB could be an imminent threat. Thus, identification of the mechanisms by which resistance to AMB can arise is an important priority, and has led to the production of laboratory-derived AMB resistance of *Leishmania* spp [96,98,99,100]. Similarly, resistance can be developed for LAMB as showed by a relapse rate around 3.7% described after treatment with liposomal amphotericin. However, despite this relatively low risk, it is important in the context of transmission dynamics, due to: a) Relapse contributes to the global pool of parasites in the host, ready for transmission to the vector; b) in HIV-positive individuals without antiretroviral therapy, VL relapse increases the risk of transmission by suppressive immunity, increased parasite burden, and lack of responsiveness to drug treatment; c) the possibility of parasite resistance to antileishmanial drugs in patients with relapsed HIV co-infection that may be a long-term reservoir resistant parasites or an increased risk of developing PKDL [101]. 

#### Resistance-Associated Mutations

No effective resistance to AMB has been reported until the moment. To anticipate a possible emergence of resistance several tests have been developed in the laboratory. Several AMB-resistant *Leishmania spp* promastigotes were selected by increasing drug pressure and were studied. The biological features of these resistant strains were compared with those of the wild-type parent strain and several mutations were observed [99]. It was demonstrated that a role for mutation in the sterol biosynthesis enzyme, lanosterol 14α-demethylase (CYP51), in a *L. mexicana* line. Genetic changes in multiple AMB-resistant *Leishmania* lines were also observed in two sterol biosynthesis enzymes: Sterol C24-methyltransferase (SMT), which introduces the C24-methyl group within the ergosterol side chain, and sterol C5-desaturase (SC5D) which is required for generation of sterol 5(6)-7(8) double bond conjugation [100].

Overall, the resistance can be attributed to the reduced AMB binding to the membrane due to an altered sterol profile (loss of function of the SMT gene). AMB is then effluxed out by the membrane-bound MDR1 and the remaining intracellular AMB auto-oxidizes and produces ROS. The toxic effect of this ROS may be neutralized by the tryparedoxin cascade of the thiol metabolic pathway. These cumulative effects of a changed membrane profile, involving MDR1 and the tryparedoxin cascade may be responsible for making the *Leishmania* parasites resistant to AMB [96,98].

### 4.4. Resistance to Miltefosine

Miltefosine (hexadecylphosphocholine, MT) is an alkyl phospholipid originally developed as an oral antineoplastic agent. MT was approved in 2002 as the first oral treatment of VL in India. Currently, MT is used to treat VL and CL diseases and is the first choice for oral treatment in CL. It has greater accessibility and presents lower toxicity compared to antimonials [63,65,79,102]. This drug shows secondary effects as hepato- and nephrotoxicity. The major limitations of MT are the teratogenicity nature, the potential of resistance due to its long half-life (~one week) and prolonged presence of sub-therapeutic concentrations, and its elevated cost [79]. 

MT inhibits the biosynthesis of phosphatidylcholine, thus affecting phospholipid biosynthesis [79,103]. The proposed mechanism of action firstly involves the binding to cell membrane, followed by the internalization through two membrane proteins: *L. donovani* miltefosine transporter (*Ld*MT), a member of the P4-ATPase subfamily; and a potential noncatalytic β subunit of *Ld*MT (*Ld*Ros3) [97,104]. Both proteins are primarily localized in the *Leishmania* plasma membrane and are required for the rapid intracellular uptake of alkylphosphocholine drugs. *Ld*MT and *Ld*Ros3 form a stable protein complex, which facilitates the translocation of phospholipids from the exoplasmic sites to the cytoplasmic sites of the plasma membrane [105]. It was also verified that MT provokes inhibition of the mitochondrial cytochrome *c* oxidase, leading to the decrease of oxygen consumption rate and ATP levels of *L. donovani*. Furthermore, it was observed that MT could induce cell death in promastigote stage of *L. donovani* by an apoptosis like process besides several immunologic and inflammatory effects on macrophages [97,102,106]. 

Despite the recent introduction of MT in the field, clinically resistant parasites have been reported in Nepal in case of VL. Induction of in vitro resistance was performed previously in the laboratory to anticipate the emergence of resistance to MT and to characterize the resulting mutants [97,107,108].

#### Resistance-Associated Mutations

Parasites with reduced susceptibility towards MT have been reported in the Indian subcontinent [109]. In 2017, two cases of VL with confirmed MT resistance (*L. donovani*) in the laboratory allowed phenotypic and genotypic characterization of the isolates [105]. Despite the rare number of MT resistant clinical isolates, their genomic and molecular profiles present high similarity to those of strains experimentally selected in the laboratory [110].

The exact mode of MT resistance is not well known, but a decrease in drug accumulation has been reported for all miltefosine resistant *Leishmania* lines studied. This result may reflect a decreased drug uptake, increased efflux, faster metabolism, or altered plasma membrane permeability. The inactivation of the transporter protein *Ld*MT is achieved with only a single point mutation within the two alleles of the *Ld*MT gene. The mutations L856P, T420N, and L832F in *Ld*MT gene demonstrated an increased rate of resistance (in vitro and in vivo) and a decreased uptake, increased efflux, faster metabolism, and changes in the lipid composition of the parasite membranes. Other mutations include V176D, W210, the recently described Y354F and F1078Y in the *Ld*MT gene, and mutation M1 in LdRos3 [105]. 

Another mechanism described for MT resistance is the overexpression of ABC transporters. LMDR1/ABCB4, a P-glycoprotein-like transporter included in the *Leishmania* ABC transporters, was the first molecule shown to be involved in experimental MT resistance. The overexpression of ABC transporters ABCB4(MDR1), ABCG4, and ABCG6 is described to be associated with an increased resistance to MT in *Leishmania*, leading to a reduction in intracellular accumulation due to increased efflux of the drug across the plasma membrane [70,73,111,112]. In addition to that, alterations in lipid compositions of membranes and sterol biosynthesis in MT-resistant *L. donovani* promastigotes could also affect drug-membrane interactions. Recently, using cosmid-based functional cloning coupled with next-generation sequencing genes involved in ergosterol biosynthesis and phospholipid translocation were suggested to contribute to resistance in *L. infantum* [70,113]. 

Several compounds have been developed to overcome the MT resistance. It was observed that sesquiterpenes can overcome multidrug resistance in *Leishmania* including resistance to miltefosine, by increasing intracellular drug concentration through the modulation of new ABC transporter activity [74]. Another flavonoid derivative in suboptimal doses showed to overcome the overexpression of LMDR1 [70]. Sitamaquine, an 8-aminoquinoline overcomes LMDR1-mediated miltefosine resistance through the increase of intracellular drug accumulation, confirming that sitamaquine can be considered to be an effective reversal agent of LMDR1-mediated miltefosine resistance in *Leishmania* parasites [111].

### 4.5. Resistance to Paromomycin

Paromomycin (PMM) is an aminoglycoside antibiotic that was introduced for the treatment of VL in 2006. PMM is usually well tolerated, can be administrated by oral route or by intramuscular injection, and secondary effects are rare [114].

PMM inhibits protein synthesis, in bacterial infections, by interacting with the ribosomal subunits and promotes association of the ribosomal subunits. It was observed the binding to the major groove in the A-site of 16S rRNA in *E. coli* and induction of misreading of mRNA. However, in the case of *Leishmania* its mode of action of is not completely perceptible. PMM in *Leishmania* parasites may act by inhibition of protein synthesis through the binding to 16S ribosomal subunit, provoking a local conformational change in the A site of 16S ribosomal RNA. The modifications at the N1 positions of A1492 and A1493 on the minor groove side of the A-site RNA implied a mechanism of action that occurred during translation. Several other mechanisms of action were proposed as: (a) Modification of membrane fluidity and lipid metabolism; (b) decreasing of the mitochondrial membrane potential; and c) respiratory dysfunction [115,116,117].

The uptake of PMM is made by endocytosis and facilitated by the binding of PMM to a number of parasite surface proteins such as paraflagellar rod (*PF*R) 1D and 2D, prohibitin, and a P-type H^+^ ATPase, whose main role is to promote endocytosis and help to enter/keep the drug inside vacuoles [94].

PMM presents several advantages as, low cost, short duration of administration, good safety profile, and accessibility. However, the physicochemical nature of paromomycin prevents enough concentration at the site of infection. The use of solid lipid nanoparticles as delivery system for PMM, demonstrated an increased penetration of the drug into the macrophages, enhancement of the immunity response, and consequently an improvement of the effectiveness of PMM [118]. A formulation consisting of albumin microspheres loaded with PMM was developed to target *Leishmania* parasites in macrophages in the treatment of VL. This formulation presents the advantages of directly target macrophages leading to decreased toxicity and being less painful than intramuscular injection [119]. PMM was also conjugated with stearylamine (SA)-bearing phosphatidylcholine (PC) liposomes. PMM-SA–PM showed an increased protective immunity effect, an excellent antileishamanial activity and no toxicity [120].

#### Resistance-Associated Mutations

PMM resistance in prokaryotes has been associated with several mechanisms, such as decreased drug accumulation, mutations at the ribosomal binding sites, and enzymatic inactivation of the drug. In eukaryotic *Leishmania* parasite the knowledge about the mechanisms of resistance is narrow, but they should involve a decreased drug uptake [73,121,122]. Several studies demonstrated that PMM resistance in *L. donovani* is associated with a decreased accumulation of drug accompanied by a reduction in the initial binding of drug to the cell surface [116]. In resistant strains it was verified a growth in the number of vesicular vacuoles and proteins involved in vesicular trafficking compared to the PMM-sensitive strain. There were also detected high levels of glycolytic enzymes, indicating that the resistant strain depends on glycolysis (aerobic or anaerobic) for its energy requirement. Stress proteins that belong to the HSP70 family, were observed with augmented basal levels relatively to the sensitive strain. 

The most reliable hypothesis of resistance suggests that after internalization of PMM by endocytosis, certain cell surface proteins are responsible for efflux the drug from the vacuole. Therefore, there are several hypotheses of PMM resistance in *Leishmania* as: (a) Modulation of translation rate; (b) interaction with vesicle-mediated trafficking; (c) an increase in energetic metabolism through glycolysis; and (d) an effective protection by chaperone/stress-related proteins [94].

## 5. Antitrypanosomal Drug Resistance

Human African trypanosomiasis (HAT) is a neglected tropical disease that occurs in sub-Saharan Africa, transmitted by the tsetse fly. HAT is characterized by two clinical variants: The West and Central African slow-progressing form, caused by *Trypanosoma brucei gambiense* (*T. b. gambiense*); and, the Eastern and Southern Africa faster progressing form, caused by *Trypanosoma brucei rhodesiense* (*T. b. rhodesiense*). Since the 1980s there was an increase in HAT cases, until 2006 when a significant reduction was reported. Nowadays, the disease burden decreased due to excellent efforts for control, elimination, or eradication by WHO and several public and public-private organizations [123,124,125]. However, HAT remains a public health problem and a fatal disease if not treated. 

*T. brucei* cells contain one central nucleus, a single mitochondrion with its own DNA, the kinetoplast and a flagellum. The life cycle of this protozoan alternates between a mammal host and an insect vector, the tsetse fly. Both gender of these flies are haematophagous and can transmit trypanosomes. Tsetse flies become infected when they ingest trypanosomes residing in the blood or in the skin of mammals. When trypanosomes enter the fly’s midgut, they undergo a series of complex changes, and multiply prior to moving to the salivary glands where the human-infective metacyclic forms are concentrated. The cycle is continued when the tsetse fly bites a new human or animal host. 

HAT develops into two major stages. An acute bloodstream and lymphatic stage, lethal to the host, where liver, spleen and heart are affected. The trypanosomes had also the ability to cross the blood-brain barrier and enter into the central nervous system, originating the second stage or the so-called “encephalitic stage” of the disease. In this stage, several neurological damages are observed as meningoencephalitis. In the case of *T.b. rhodesiense* this stage can occur after few weeks and in the case of *T. b. gambiense* can take few or several months to occur. The *T. brucei* subspecies differ in their virulence, with *T. b. rhodesiense* causing a more acute and aggressive HAT, and probably death, while the disease caused by *T. b. gambiense* develops slower. The motive that can explain the differences between the two variants is not clear. The parasite cell membrane in the mammalian host is covered by highly immunogenic glycoproteins that can induce a specific antibody response that causes destruction of all parasites opsonised with these antibodies. In a way to resist to this antibody-mediated immune response, parasites developed an “antigenic variation”, in which the glycoprotein coat on the cell membrane is replaced by an antigenically different coat. The interaction between immune response of the host and antigenic variation of the parasite results in an irregular parasitaemia, reflected by irregular fevers [123,126]. 

**Figure 3:**
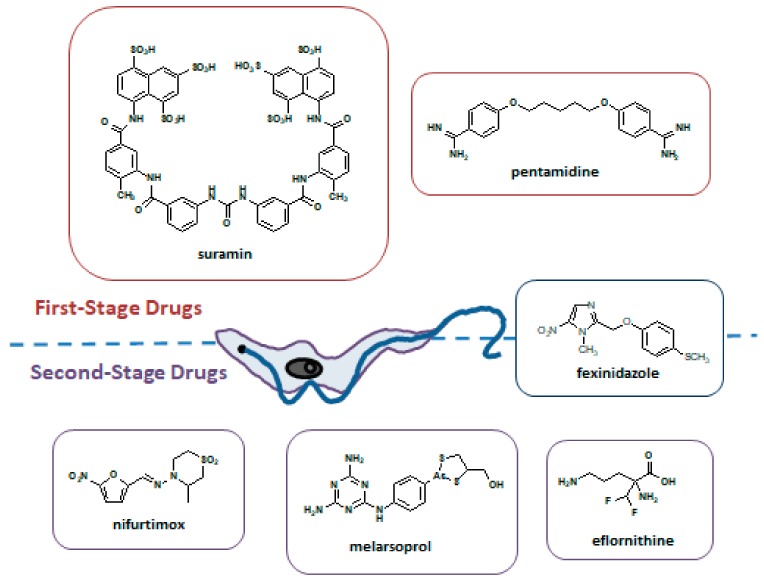
The recommended drugs in the treatment of trypanosomiasis. Chemical structures and stages of the disease where they are used.

The type of treatment depends on the disease stage. While the administration of safer drugs is preferred in the first stage of infection, the second stage requires drugs capable of crossing the blood-brain barrier to reach the parasite. Such drugs are generally toxic and complicated to administer. These protozoa trypanosomes may remain inactive for long time in host and give origin to the disease months after treatment. For this reason, there is the need to perform routine analysis to body fluids, including cerebrospinal fluid, and to follow up the patient up to 24 months after treatment. Currently five drugs are in use for the treatment of HAT (Figure 3). 

For the first stage of *T. b. gambiense* and *T. b. rhodesiense* the drugs recommended are pentamidine and suramin. Since 1990 eflornithine has been used as monotherapy to treat the second stage of *T. b. gambiense*. Its use is currently recommended as part of NECT (nifurtimox-eflornithine combination therapy). Although the efficacy of NECT was similar to that exhibited by eflornithine monotherapy, NECT reduced treatment failure and eflornithine dosage [127,128,129]. Melarsoprol, in use since 1949, besides highly toxic and with resistance cases reported, is the only available treatment for the second stage of *T. b. gambiense* and *T. b. rhodesiense* in East Africa. 

In 2018, fexinidazole received a positive opinion from the European Medicines Agency (EMA) for the treatment of first-stage and second-stage of *T. b. gambiense* HAT (*g*-HAT) in adults and children under six years and weighing ≥ 20 kg, supporting the ongoing registration process in endemic countries and further distribution by the WHO [130].

Despite the good efforts to control HAT, there is an urgent need for new drugs, mainly due to the emergence of resistance to pentamidine and melarsoprol that have been used for the treatment of African trypanosomiasis for decades. An understanding of the mechanisms of resistance, and particularly of cross-resistance, is of great importance [131]. A summary of drugs used for the treatment of HAT is presented in Table 3.

The protozoa trypanosomes have the amazing ability to adapt to drug pressure, being extremely versatile organisms. The cases of drug resistance may be due to: (a) Low drug levels inside the cell (influx/efflux ratio); (b) alteration of the molecular target of the drug (associated with loss of activity); and (c) general defense and repair mechanisms. The rate of drug resistance development is lower compared to other infectious diseases because, with the exception of eflornithine, all drugs used to treat trypanosomiasis exhibit a multitarget effect. It is worth noting, that higher levels of drug resistance in rural and endemic areas can be justified by the zoonotic characteristics of the disease and by the indiscriminate and incorrect use of chemically similar drugs in humans and animals [127]. 

### 5.1. Resistance to Pentamidine

Pentamidine (PMD) also currently in use to treat leishmaniasis, was first discovered for trypanosomiasis treatment and it continues in therapeutic until nowadays, being well tolerated by patients despite non-negligible undesirable effects. Pentamidine has been used to treat the first stage of HAT and is more effective against *g*-HAT than *T.b. rhodesiense* HAT (*r*-HAT). 

Several transporters as the P2 adenosine/adenine may contribute to PMD uptake in trypanosomes, and after internalization by endocytosis, PMD binds to the receptor aquaglyceroporin 2 (AQP2) with affinity in the nM range [127]. The intracellular accumulation of PMD in DNA containing compartments, as the nucleus and the mitochondrion is facilitated by active transport and endocytosis. Pentamidine acts as a DNA-binding-drug, provoking the collapse of the parasite mitochondrial membrane potential, and the induction of kinetoplast DNA destruction [129,131,132]. 

#### Resistance-Associated Mutations

Uptake of PMD in *Trypanosoma brucei* spp. is probably mediated by the P2 aminopurine transporter and loss of function of this transporter has been implicated in resistance to this agent. The pentamidine/melarsoprol cross-resistance is a major concern for HAT treatment [127]. In addition to the AT1/P2 transporter, the other surface protein involved in pentamidine/melarsoprol cross-resistance is AQP2, whose main function is related with osmoregulation. [133].

### 5.2. Resistance to Suramin

Suramin is a polysulphonated symmetrical naphthalene derivative highly effective for the treatment of the first stage of *T.b. rhodesiense* infection. Suramin presents synergism with the second stage drugs eflornithine, nifurtimox, and melarsoprol. In contrast, suramin inhibits the activity of pentamidine [134].

Glycolysis has been proposed as the most likely target. However, other pathways could be targeted by the drug. For example, suramin is a competitive inhibitor of 6-phosphogluconate dehydrogenase, an enzyme of the pentose phosphate pathway, but the mode of action is not exactly known. The hypothesis of several enzymes involved in the mechanism of action may be an explanation for the lack of resistance in humans for suramin [134,135]. More recently, suramin was found to inhibit cytokinesis, since *T. brucei* cells with more than two nuclei were observed when in contact with the drug, indicating a defect in cytokinesis with continued mitosis [129].

In addition to being in use for almost a hundred years and a half-life of around 44–54 days, there have been no reports of suramin resistance in human pathogenic trypanosomes. Suramin resistant strains were obtained under laboratory conditions. It was verified that a variant surface glycoprotein (VSG) that cover the bloodstream-form trypanosomes, and that protects them from their mammalian hosts’ immune defenses, might be involved in the emergence of resistance. After exposure to high suramin concentrations the gene VSG^Sur^ was expressed and correlated with suramin resistance [135].

### 5.3. Resistance to Melarsoprol

Inorganic arsenical compounds have been extensively used and the first recorded use in the treatment of trypanosomiasis was in 1858. Discovered in 1949, melarsoprol (Mel B) it is currently used for the treatment of both *gambiense* and *rhodesiense* infections and is administrated intravenously. It has a high number of undesirable side effects being the most dramatic of which a reactive encephalopathy that can be fatal. It is currently recommended for the first stage treatment of *r*-HAT, and for the second stage of *g*-HAT, but due to the severe side effects the nifurtimox-eflornithine combination therapy (NECT) has largely supplanted Mel B in West Africa for the treatment of *g*-HAT. Mel B is still in use today in East Africa as the only effective treatment for advanced stage *r*-HAT [129]. The only drugs that can cross the blood-brain barrier and be used in the second stage of the disease are Mel B and NECT, with Mel B being the only one with clinically proved resistance, particularly in central Africa [136]. 

Mel B is a trivalent arsenical compound and the mechanism of action is not completely clear. It has affinity for sulfhydryl groups, in particular for vicinal thiols in proteins, but this is a moderately nonspecific effect that lack cellular selectivity between host and parasite. The selective uptake of Mel B is made by the transporters P2 adenosine (AT1 gene) and aquaglyceroporin 2 (AQP2) of the parasite. Once in the cell, it reacts with the dithiol group of trypanothione forming a complex which is a competitive inhibitor of the trypanothione redutase. However, there is no definitive evidence that this mechanism leads to the cell death. Alternatively, cell lysis might be related to the glycolytic pathway of the parasite. More recently it was observed that Mel B inhibited mitosis and that this mitotic defect is dependent upon a specific set of kinases [129,136]. 

#### Resistance-Associated Mutations

Loss of drug uptake is the major mechanism of drug resistance in trypanosomes. Mutations in both transporters P2 and AQP2 are responsible for low uptake inside the cell. Melarsoprol-pentamidine cross resistance (MPXP) exists for HAT since both drugs share the same transporters, particularly AQP2. The resistance to Mel B is related to mutations in adenosine transporters P2 as well as mutations at the chimeric aquaglyceroporin AQP2–AQP3 [137]. 

### 5.4. Resistance to Eflornithine

Eflornithine, (α-difluoromethylornithine, DFMO) is currently the only treatment available for the second stage of *T. b. gambiense* HAT when there is no response to melarsoprol. It is used in combination with nifurtimox and represents a major advance in terms of safer, cheaper, and easier to administer treatment [138].

The mode of action of DFMO is related with the selective and irreversible inhibition of ornithine decarboxylase (ODC), an enzyme essential in the biosynthesis of the polyamines spermidine and spermine, involved in nucleic acid synthesis and regulation of proteins. The slow turnover of ODC in *T. b. gambiense* is responsible for the selectivity of DMFO between humans and parasite cells. Not surprisingly, the lack of susceptibility of *T. b. rhodesiense* to DFMO has been ascribed to the higher ODC turnover in this parasite [139]. The risk of clinical resistance to DMFO is, unfortunately, a reality due to their continued administration alone or in combination with nifurtimox [140]. 

#### Resistance-Associated Mutations

The transporters were found to be crucial for sensitivity and resistance to drugs. Resistance to eflornithine was easily selected in the laboratory and must be related to amino acid transporters. In cases of resistance it was found that the gene of the amino acid transporter TbAAT6 was absent and that was responsible for low uptake of DMFO and loss of sensitivity [140,141]. 

### 5.5. Resistance to Nifurtimox

Nifurtimox, Nfx, is a nitrofuran and presents activity against African and American (i.e., *T. cruzi*, responsible for Chagas´ disease) forms of trypanosomiasis. The nifurtimox efficacy for the chronic forms of the disease appears to be geographically dependent, with better results in Chile, Venezuela, Argentina, and southern Brazil than in central Brazil [142]. Nfx is only registered for American trypanosomiasis and the administration was only allowed in Africa with authorization and acceptance of responsibility by national authorities. However, after safety and efficacy data, since 2009 its use is recommended in combination with DMFO (NECT) for the treatment of *g*-HAT. Both drugs are provided free of charge by WHO to endemic countries with a kit containing all the material needed for its administration. NECT consists of Nfx delivered orally and DMFO delivered intravenously [126].

Nitroheterocyclic compounds as Nfx are prodrugs and require activation to mediate cytotoxic activity. The mode of action of Nfx is poorly understood, but a possibility involves reductive activation via an NADH-dependent bacterial-like nitroreductase (NTR) with the formation of a cytotoxic, unsaturated open-chain nitrile derivative. The one-electron reduction of the nitro group can also generate free radicals that can induce oxidative damage, leading to death of the parasite [143,144]. Recently it was found that Nfx provokes a severe disruption of mitochondrial structure and function, consistent with damage of targets in the organelle where the drug is activated inducing a specific reduction in mitochondrial protein content [129].

#### Resistance-Associated Mutations

Concerted efforts were made to elucidate the potential mechanisms of drug resistance of Nfx. It was verified in laboratory-generated clones of *T. cruzi* that resistance is associated with loss of NTR activity [145]. The resistance mechanism to Nfx in *T. brucei* has been assessed and mainly six genes were directly/indirectly related with the dominant role of the NTR in activation of Nfx being associated with resistance [145]. 

### 5.6. Resistance to Fexinidazole

Fexinidazole (FEX), is a 2-substituted 5-nitroimidazole identified in the late 1970s as a broad-spectrum anti-infective agent, including for HAT. FEX showed potent activity against *T. brucei* in preclinical assays, but due to lack of commercial viability, it was abandoned. Almost 30 years later, the same compound was rediscovered as a hit upon screening of a nitroheterocyclics library by the Drugs for Neglected Diseases initiative (DNDi) [146]. The approval by the EMA in 2018 facilitated and supported marketing authorization application in endemic countries in 2019. 

FEX is the only available oral monotherapy developed and tested, so far, to treat patients with late-stage g-HAT. It is also appropriate for both the first and second stage of the disease. Another advantage is the easier administration, reducing the need for patient hospitalization, lumbar punctures, and all the potential complications associated with intravenous catheter use, which may also have a positive pharmacoeconomic impact. Currently, a Phase 3 evaluation of FEX for *g*-HAT is ongoing in the Democratic Republic of the Congo and Guinea, and the drug is also in development for Chagas’ disease, with a study ongoing in Spain [130]. WHO is updating treatment guidelines that will define the role of FEX in the current therapeutic armamentarium. 

The exact mechanism of action of FEX and its two metabolites (FEX sulfoxide or FEX sulfone) is unknown, but it has been reported that this drug can inhibit DNA synthesis [130]. However, and similarly to Nfx, FEX requires bioactivation by NTR in order to exert its selective activity against the parasite [147]. The mechanism of action for Nfx and FEX based on the same NTR enzyme potentiates the possibility of cross-resistance which represents a major disadvantage [147]. In the laboratory it was observed that the mechanisms of resistance to FEX were the same presented for Nfx [145]. 

## 6. Perspectives

Resistance to antimalarial drugs remains a major threat to the global efforts to control and eliminate malaria. Despite improved access to effective malaria treatments has decisively contributed to the significant reduction in the malaria burden, recent reports reveal that, after an unprecedented period of success in global malaria control, the reduction of clinical cases has come to a stall. Failure of leishmaniasis treatment is also becoming an increasing problem exacerbated by the limited therapeutic options, associated to toxicity, high cost, and growing drug resistance. The situation in trypanosomiasis-affected regions is not different, where current therapies are characterized by high toxicity and increasing drug resistance associated, at least in part, with loss-of-function mutations in the transporters involved in drug import. 

Protecting the efficacy of the recommended malaria, leishmaniasis, and trypanosomiasis treatments is critical for endemic countries. The scientific community continues to be strongly committed to overcome the emergence and spread of parasites resistant to existing drugs. However, long-term strategies are required to attain the ultimate goal of eliminating these parasitic diseases. These strategies should include a better understanding of the mechanisms of action and resistance of clinical candidates and compounds already in more advanced stages of clinical trials, in order to design alternative and safer molecules. Furthermore, new molecules acting on new unprecedented targets are urgently needed to increase the chemotherapeutic arsenal, overcome safety limitations, and counter the emerging resistant parasites. Public-private partnerships are perhaps the best equipped platforms to support R&D on new drugs at a sustainable scale, providing preclinical research expertise, and technical support related to early-stage antibiotic drug discovery and product development. Hopefully, these combined efforts will deliver the desired outcomes in a near future.

## Figures and Tables

**Table 1:** Antimalarial drugs, their uses, and mechanisms of resistance.

Drug	Use in [12]	Mode of Action	Mechanism of Described Resistance
Chloroquine	Uncomplicated non-falciparum malaria	Inhibition of heme detoxification	*Pf*crt, *Pf*mdr1, *Pf*mrp1, *Pf*nhe, *Pf*ATP4 mutations
Amodiaquine	Uncomplicated *P. falciparum* or *P.vivax* infections in ACT Chemoprophylaxis with SP		
Quinine	Severe and uncomplicated malaria Alternative when effective ACT is not available		
Mefloquine	Uncomplicated malaria in combination with artesunate Chemoprophylaxis of malaria caused by all species		
Primaquine	Radical cure of *P. vivax* or *P. ovale* Anti-relapse therapy for P. *vivax* and P. *ovale* Gametocytocidal agent	Possibly a unique mode of action involving CYP2D6 and CPR [13]	
Lumefantrine	Treatment of uncomplicated malaria (all species) in combination with artemether	Inhibition of heme detoxification	*Pf*crt, *Pf*mdr1, *Pf*mrp1 mutations
Sulfadoxine	SP for the treatment of malaria in pregnant women and children SP in combination with amodiaquine for seasonal chemoprevention in children acute uncomplicated malaria in combination with artesunate	Competitive inhibition of *Pf*DHPS	*Pf*dhps, *Pf*dhfr-ts mutations
Pyrimethamine		Inhibition on folate biosynthesis (*Pf*DHFR)	
Proguanil			
Atovaquone	Prophylaxis of malaria and treatment of uncomplicated malaria in travellers outside endemic areas in combination with proguanil	Inhibits the respiratory function of parasite	*Pf*cytb mutation
Artemisinin Artesunate Artemether	Multidrug-resistant *Pf* infection Combination with other drugs to prevent drug resistance (ACT) Children and adults with uncomplicated *P. falciparum* malaria and severe malaria	Generation of free radicals and reactive species and alkylation of parasite target biomolecules	
			*Pf*K13

(ACT—Artemisinin Combination Therapy; SP—Sulfadoxine-Pyrimethamine; DHPS—Dihydropteroate synthetase; DHFR—dihydrofolate reductase; CPR—cytochrome P450 NADPH:oxidoreductase).

**Table 2:** Antileishmanial drugs, their uses, and mechanisms of resistance.

Drug	Use in	Mode of Action	Resistance Described	Mechanism of Resistance
SodiumStibogluconate	All clinical forms of leishmaniasis Combination therapy (with PMM)	Trypanothione reductase Inhibition	Yes	Elevated intracellular thiols levels Overexpression of: TXNPx, MRP1, and ABC transporters
Pentamidine	Systemic CL Secondary prophylaxis of VL treatment in HIV co-infection	Not clear. Hypothesis: Interaction with kDNAs; interference with polyamine synthesis; inhibition of RNA polymerase; inhibition of TOPII; apoptotic death	Yes	Overexpression of PRP1AQP2 mutation
Amphotericin B and Liposomal Amphotericin B	VL Combination therapy (with MT and PMM)	Not clear. Hypothesis: Apoptotic death, depolarization of the membrane	No effective resistance	Several hypotheses based on laboratory-derived resistant strains
Miltefosine	VL, CL, combination therapy (with LAMB)	Not clear. Hypothesis: Alteration in alkyl-lipid metabolism and phospholipid biosynthesis, apoptotic death	No effective resistance	Several hypotheses based on laboratory-derived resistant strains
Paromomycin	CL, PKDL, combination therapy (with SSG, LAMB and MT)	Not clear. Hypothesis: Inhibition of protein synthesis, decreasing of mitochondrial membrane potencial, alteration in membrane fluidity and lipid metabolism, respiratory dysfunction	No effective resistance	Several hypotheses based on laboratory-derived resistant strains

(kDNA—kinetoplast DNA; TOP II—topoisomerase II; SSG—sodium stibogluconate; LAMB—Liposomal amphotericin B; MT—miltefosine; PMM—paromomycin).

**Table 3:** Drugs for HAT treatment, their uses, and mechanisms of resistance.

Drug	Use in	Mode of action	Resistance Described	Mechanism of Resistance
Pentamidine	*g*-HAT and *r*-HAT 1^st^ stage	Interferes with the nuclear mechanisms, inhibiting synthesis of DNA, RNA	Yes	Loss of function of P2 aminopurine transporter
Suramin	*g*-HAT and *r*-HAT 1^st^ stage	Inhibition of glycolytic enzymes	No effective resistance	Several hypotheses based on laboratory-derived resistant strains
Melarsoprol	*g*-HAT and *r*-HAT 2^nd^ stage	Not completely clear	Yes	Mutations in P2 and AQP2 transporters
Eflornithine	*g*-HAT 2^nd^ stage Used in combination with nifurtimox (NECT)	Inhibition of ornithine decarboxylase, an enzyme involved in polyamine synthesis in trypanosomes	No effective resistance	Several hypotheses based on laboratory-derived resistant strains
Nifurtimox	*g*-HAT 2^nd^ stage Used in combination with eflornithine (NECT)	Inhibition of trypaniothione reductase, generation of free radicals toxic for the trypanosome, and mitochondrial disruption	No effective resistance	Several hypotheses based on laboratory-derived resistant strains
Fexinidazole	*g*-HAT 1^st^ stage and 2^nd^ stage	DNA synthesis inhibitor	No effective resistance	Several hypotheses based on laboratory-derived resistant strains

(*g*-HAT—T. b. gambiense HAT; *r*-HAT—T.b. rhodesiense HAT).
